# Pulmonary Vein Stenosis in a Newborn: A Commonly Overlooked Diagnosis

**DOI:** 10.1155/2015/870257

**Published:** 2015-09-20

**Authors:** Nathalie Jeanne Magioli Bravo-valenzuela, Guilherme Ricardo Nunes Silva, Marcela Pinto Varella

**Affiliations:** ^1^Pediatrics Department, University of Taubaté, 12020-130 Taubaté, SP, Brazil; ^2^University of Taubaté, Taubaté, SP, Brazil; ^3^Hospital of Taubaté University, Avenida Granadeiro Guimaraes 270, Centro, 12020-130 Taubaté, SP, Brazil

## Abstract

The diagnosis of primary pulmonary vein stenosis is often overlooked because its symptoms overlap lung diseases and pulmonary arterial hypertension. Its diagnosis may be difficult because the condition is progressive and associated with other defects. We present a case of pulmonary vein stenosis in a newborn with stenosis of the left-sided common pulmonary vein, diffuse hypoplasia of the superior right pulmonary vein, and atresia of the inferior right pulmonary vein.

## 1. Introduction

Pulmonary vein stenosis is a rare condition with high morbidity and mortality and a frequency of 1.7 among 100,000 children less than 2 years of age [1]. Primary pulmonary vein stenosis develops from an abnormal developmental process, most likely because of the abnormal incorporation of the pulmonary veins into the left atrium (LA) [2]. Approximately 50% of patients with primary pulmonary vein stenosis also have other congenital heart defects (CHD), most commonly atrial and ventricular septal defects [2]. Secondary pulmonary vein stenosis can develop because of external compression, cardiac catheterization, or surgical repair [1, 3].

Clinical profile of pulmonary vein stenosis varies with the number of pulmonary veins involved and the severity of the stenosis, which can be discrete (shelf type), can affect a longer segment, or can be diffuse, causing severe pulmonary vein stenosis or atresia. Primary pulmonary vein stenosis is generally progressive and has been associated with neoproliferation of myofibroblast cells [3]. Clinical presentation includes progressive tachypnea and pulmonary edema shortly after birth. Pulmonary arterial hypertension predominates as stenosis progresses.

We report on a rare case of a newborn with stenosis of the left-sided common pulmonary vein, diffuse hypoplasia of the superior right pulmonary vein, and atresia of the inferior right pulmonary vein.

## 2. Clinical Presentation

A full-term newborn male (2345 g) presented craniofacial malformations with early respiratory distress and central cyanosis. At birth, the cyanosis did not improve even after oxygen administration (90% and 84% arterial saturation by pulse oximetry in upper and lower right limbs, resp.). His prenatal ultrasound showed various malformations (cleft palate, mandibular hypoplasia, and ventricular septal defect).

Blood pressure and peripheral pulses were normal. Cardiac auscultation detected accentuation of the pulmonary component of the second heart sound and systolic murmur grade 2/6 at the lower left sternal border. The electrocardiogram and chest X-ray revealed right atrial/ventricular enlargement and cardiomegaly, respectively.

Initial transthoracic echocardiography showed an ostium secundum atrial septal defect (4 mm), perimembranous ventricular septal defect (7 mm), patent ductus arteriosus (PDA), and severe pulmonary hypertension (right-to-left shunt across the atrial septum and PDA). The patient received selective pulmonary vasodilators, sympathomimetic amines, furosemide, and mechanical ventilation. Although G-banded karyotyping was normal, Pierre Robin Sequence was suspected.

The patient's clinical condition and arterial saturation worsened. The second chest X-ray suggested asymmetric pulmonary vascularity (discrete increased blood flow in the left lung). The follow-up transthoracic echocardiogram suggested a membrane in the left atrium and showed worsening of the pulmonary arterial hypertension. The flow of the pulmonary veins in the left atrium was not visualized.

**Figure 1:**
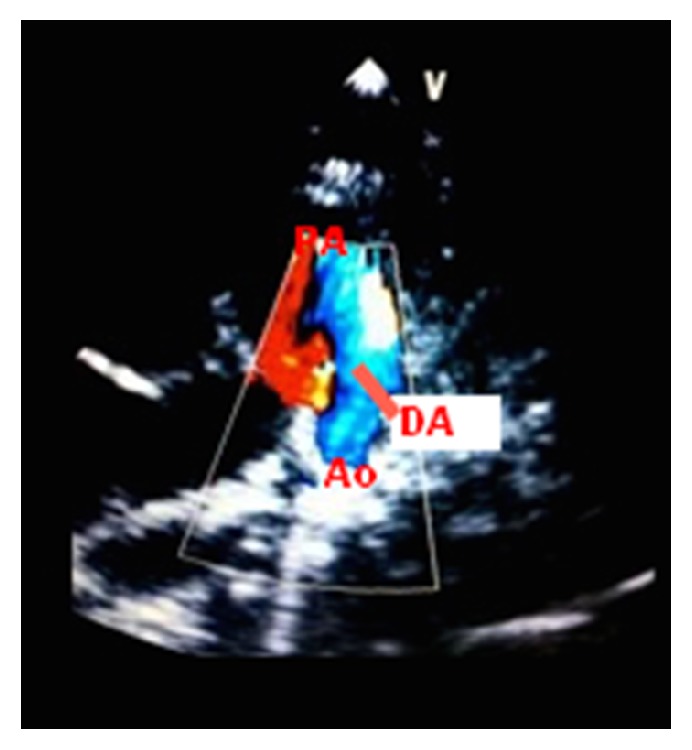
Transthoracic echocardiogram showing patent ductus arteriosus with flow (blue) from the pulmonary artery to the aorta. PA: pulmonary artery, DA: ductus arteriosus, and Ao: aorta.

**Figure 2:**
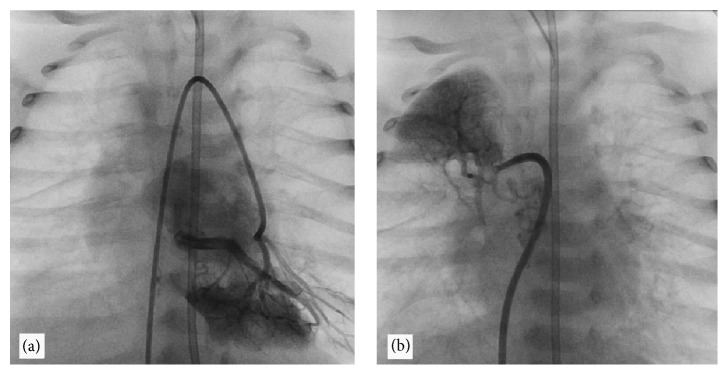
Cardiac catheterization after injection in (a) and (b) pulmonary arteries: (a) common pulmonary vein with stenosis at the connection point draining into the left atrium and (b) right lower PV atresia with drainage through the collateral network to the right vertebral vein and azygos vein and right upper PV with severe segmental hypoplasia; PV: pulmonary vein.

The reversed shunt (right-to-left) across the PDA and estimated right ventricular systolic pressure by tricuspid regurgitation (70 mmHg) were pertinent to the echocardiographic diagnosis of pulmonary arterial hypertension (Figure 1). The heart defects with unexplained progressive pulmonary hypertension suggested pulmonary vein stenosis. Cardiac catheterization confirmed the pulmonary arterial hypertension (pressure: right ventricle = 70/15 mmHg, aorta = 70/45 mmHg, and left/right atria = 10 mmHg). Angiography revealed that the left pulmonary veins drained into the LA through a common pulmonary vein with stenosis at the connection point, atresia of the lower right pulmonary vein (drainage through the collateral network), and severe segmental hypoplasia of the upper right pulmonary vein (Figures 2(a) and 2(b)).

The surgical correction was performed by marsupialization technique and the atrial septal defect was maintained to alleviate pulmonary arterial hypertension. After four months, restenosis occurred, percutaneous balloon dilation was attempted without success, and the patient died.

## 3. Discussion

Developmental abnormalities of the pulmonary venous plexus and common pulmonary vein absorption into the LA as well as the atresia or stenosis of the common pulmonary vein result in a spectrum of pulmonary venous anomalies. The LA and pulmonary venous system develop separately. The latter originates from blood vessel beds of the splanchnic system. Initially the common pulmonary vein empties into the LA and then four independent pulmonary veins are incorporated into the atrium [2].

Failure of the incorporation of the common pulmonary vein into the LA is the most accepted theory of embryogenesis of Cor triatriatum sinister. In Cor triatriatum sinister, the LA is divided into two chambers (proximal and distal) by a fibromuscular membrane, causing symptoms of venous obstruction that should be distinguished from pulmonary venous stenosis. The noninvasive diagnosis can be made by echocardiography; however, it may be hindered in case of Cor triatriatum sinister combined with pulmonary vein anomalies [3].

Total anomalous pulmonary venous drainage, particularly the infracardiac type, can mimic respiratory distress of different etiologies during the neonatal period. The echocardiographic clues to this diagnosis are (1) identification of the vertical vein (because obstruction can be at this level), (2) presence of atrial septal defect with right-to-left shunt, (3) enlarged right heart chambers, (4) small LA, and (5) inability to image the pulmonary venous draining into the LA [4]. Total anomalous pulmonary venous drainage, particularly the infracardiac type, is associated with pulmonary venous obstruction and can mimic other etiologies of respiratory distress and hypoxia during neonatal period. Atresia of the common pulmonary vein is a rare and obstructive form of this condition [5]. In this study, the common pulmonary vein was stenosed instead of being atresic and difficult to be visualized by transthoracic echocardiogram. Therefore, when echocardiography fails to identify pulmonary venous return, CT angiography, magnetic resonance, or cardiac catheterization should be employed [6].

Unilateral pulmonary vein atresia with contralateral pulmonary vein stenosis is an extremely rare congenital defect with high morbidity and mortality. This diagnosis should be suspected particularly in cases of lung congestion and atypical evolution of pulmonary arterial hypertension [7].

Based on echocardiography, the diagnosis of pulmonary vein stenosis can be suspected by visualization of the pulmonary veins connected to the LA with a turbulent flow on color Doppler. Pulsed Doppler with monophasic pulmonary venous flow and peak velocities >1.6 m/s are important findings of pulmonary vein stenosis. If these findings are not observed, follow-up echocardiograms should be performed, focusing on color-flow mapping of all pulmonary veins [7].

The mortality rates for patients with pulmonary vein stenosis are high even after surgery [1, 3]. Some centers prefer the marsupialization technique that uses the pericardium to reconstruct the pulmonary vein [8]. Another option is lung transplantation or pneumonectomy when stenosis is unilateral. Percutaneous angioplasty temporarily relieves stenosis or complements surgery, and cutting balloon with large-caliber stenting (>7 mm) provides the best result [9]. However, restenosis occurs in 50% of patients one year after catheterization and within 5 years after surgery [8, 9]. In vitro studies suggested that endothelial factors (transforming growth factor-*β*1) may be involved in the restenosis genesis [10]. These findings suggest a neoproliferative process and point out future directions for research on the antiproliferative therapy [10].

Although pulmonary vein stenosis is a rare congenital condition, it should be suspected in infants presenting with the clinical features such as those in the case reported. Cyanosis with refractory hypoxemia, persistent respiratory distress, and unexplained pulmonary arterial hypertension should draw attention to this diagnosis.

## Supplementary Material

Pulmonary cineangiography video (online). Cardiac catheterization showing the dynamic images presented in Figure 2 (A and B). Left pulmonary artery angiography demonstrating the common pulmonary vein with stenosis at the connection point draining into the left atrium. Sequential right pulmonary artery angiography showing the right lower PV atresia with drainage through the collateral network to the right vertebral vein and azygos vein, and right upper PV with severe segmental hypoplasia. PV: pulmonary vein.
